# Teaching wilderness first aid in a remote First Nations community: the story of the Sachigo Lake Wilderness Emergency Response Education Initiative

**DOI:** 10.3402/ijch.v71i0.19002

**Published:** 2012-10-26

**Authors:** Karen Born, Aaron Orkin, David VanderBurgh, Jackson Beardy

**Affiliations:** 1 Institute of Health Policy & Management, Faculty of Medicine, University of Toronto, Toronto, ON, Canada; 2 Division of Clinical Science, Northern Ontario School of Medicine, ON, Canada; 3 Dalla Lana School of Public Health, University of Toronto, ON, Canada; 4 Sachigo Lake First Nation, ON, Canada

**Keywords:** first response, Aboriginal health, access to trauma care, community-based participatory research, qualitative research

## Abstract

**Objective:**

To understand how community members of a remote First Nations community respond to an emergency first aid education programme.

**Study design:**

A qualitative study involving focus groups and participant observation as part of a community-based participatory research project, which involved the development and implementation of a wilderness first aid course in collaboration with the community.

**Methods:**

Twenty community members participated in the course and agreed to be part of the research focus groups. Three community research partners validated and reviewed the data collected from this process. These data were coded and analysed using open coding.

**Results:**

Community members responded to the course in ways related to their past experiences with injury and first aid, both as individuals and as members of the community. Feelings of confidence and self-efficacy related access to care and treatment of injury surfaced during the course. Findings also highlighted how the context of the remote First Nations community influenced the delivery and development of course materials.

**Conclusions:**

Developing and delivering a first aid course in a remote community requires sensitivity towards the response of participants to the course, as well as the context in which it is being delivered. Employing collaborative approaches to teaching first aid can aim to address these unique needs. Though delivery of a first response training programme in a small remote community will probably not impact the morbidity and mortality associated with injury, it has the potential to impact community self-efficacy and confidence when responding to an emergency situation.

Elevated accident trauma rates are a documented health concern in remote First Nations communities in Canada (1). The effects of geographic isolation on timely response in many remote First Nations communities have also been reported (2,3).

Timely access to high quality trauma care has been demonstrated to save lives (4). However, for many remote First Nations communities, injury burden is magnified by a lack of timely access to first response, as well as significant distances from secondary, tertiary or quaternary health care services.

In remote First Nations communities, access to urgent care provided by physicians within a hospital setting is often available only by air transport. These communities also lack paramedic capacity. While some First Nations communities have developed teams of first responders and logistics for dealing with emergencies, there are challenges, such as limited training and logistical support for these teams.

The Sachigo Lake Wilderness Emergency Response Education Initiative (SLWEREI) was motivated by the evidence demonstrating a higher burden of trauma, coupled with the challenge of access to high quality first response in many remote communities (3). The purpose of this project is to better understand the challenges around first response and work with the Sachigo Lake community to develop and deliver a regionally and culturally appropriate training programme.

In this article, we briefly describe SLWEREI, with a focus on the question, “what is the response of a remote First Nations community to an emergency first aid course?”

Results provide insights into understanding emergencies within the remote First Nations context and describe how a course that engages community members in its development and design can help fill a knowledge and resource gap.

## Materials and methods

### Research setting

Sachigo Lake (population 400) is situated in the subarctic boreal forest of Northwest Ontario, located 425 km north of Sioux Lookout, which is a hub of transport and services for the remote reserves in the region. Like most of the 28 Oji-Cree reserves in this 3,00,000 square km region, Sachigo Lake is accessible by plane year-round or ice roads during the winter.

Basic primary care is provided through a nursing station, funded by Health Canada, which is generally staffed by 3 nurses and community health workers. A family physician, based in Sioux Lookout, visits the community for 3–4 days per month and is available for telephone consultations when not in the community. Transport times to hospital services and specialist care in Sioux Lookout, Thunder Bay or Winnipeg are seldom less than 4 hours, and with weather or other delays, transport times can be significantly longer.

### Study population

Course participation was purposive and under the discretion of the community research partner, who is the Director of the Health Authority, member of the band council and Sergeant of the local detachment of the Canadian Rangers (5). The community research partner selected course participants based on his own discretion and related to his view of who would benefit from the course. Participants were purposively selected from various backgrounds in the community and included community health workers, Canadian Rangers, educators, maintenance workers, Band Council members and employees of the community store. Some course participants had multiple roles, for example, being both a staff member at the school and a member of the Band Council. There were 20 participants in total (5% of the community population), including 3 research partners. Thirteen men and seven women participated in the course.

The course content and design is described elsewhere (6).

### Data collection and analysis

The programme design and research process employed community-based participatory research (CBPR) methodology. CBPR is a framework that can be applied to gain better understanding of the social context related to a phenomena being studied and aims to work with community partners to facilitate ownership of the research process and use the results to improve the community (7,8).

The CBPR process followed key principles of participation and equity of the community, sensitive to the needs and interests of the community partners. These principles are: (a) integration of community members, participants, research and programme delivery team as equal partners in every phase, (b) structural and functional integration of the intervention and evaluation components, (c) a flexible agenda, responsive to the demands from the broader environment and (d) create a project representing learning opportunities for all involved.

The research team met with community partners and stakeholders in May 2010, which focused on learning about community needs and past critical medical events in the community, as well as establishing relationships and trust between partners. The choice of methodology was based on input from community members, who expressed a preference for participant observation, focus groups and sharing circles. Researchers engaged in CBPR projects with First Nations communities in Canada have developed Research Agreements to codify and clarify expectations, control of data, roles and responsibilities of researchers, community research partners and community members, and to set out a mutuallyagreed upon knowledge translation and dissemination plans of the research (8). Principles for the Research Agreement were discussed during the site visit and finalised in November 2010.

At the outset of the course, the researcher conducted a short presentation outlining the key aspects of consent in plain language for the participants.

Eight focus groups, generally with 4 participants each, took place on the first and third day of the course. Each of these focus groups were approximately 45 minutes in duration. In addition, a sharing circle with all participants (n=20) took place during the final day of the course at the closing banquet. Both the focus groups and sharing circle were facilitated by an embedded researcher, who conducted participatory observation throughout the course duration.

The sharing circle is a process, where people speak in turns. Sharing circles have been used in resarch with First Nations for seeking consensus in decision-making, resolving conflicts and building trust (9). Focus groups were the preferred method of course participants, and course participants were told that focus groups were not mandatory; however, attendance was 100%. Themes were then discussed with community research partners (n=3). Community research partners included the local Health Director as well as two members of the community who participated in the course, selected by the Health Director. These individuals had no training in qualitative research; however, during meetings they reflected on key themes drawn from focus groups. Themes were discussed between the researcher, research partners and Health Director.

Focus groups are often used as a mode of data collection in research with First Nations’ communities (10). The language used to describe themes is drawn from health research and evaluation literature. For example, the term, resilience, is used to describe how First Nations individuals and communities attain positive outcomes in a climate of risk and adversity, based on the legacies of past colonialism, dislocation and exploitation (11). Similarly, self-efficacy has been defined as the belief that one can perform specific activities in specific situations. Self-efficacy is rooted in context and varies based on situations, environment and context (12). The concept of self-efficacy has been applied to similar research projects. For example, the Sandy Lake Health and Diabetes Project used a scale of dietary self-efficacy to measure the impact of a culturally appropriate, school-based dietary intervention among children in that community (13).

A semi-structured interview guide (Table I) with questions focused on the course experience was used during the focus group. As per the wishes of participants, in lieu of recording, the researcher took detailed and often verbatim notes (14).

**Table I:** Sample interview guide

Interview date	Sample questions
Courses days 1–3	Can you share a story about something that you experienced that you learned about today?
	Is there something today (from specific session) that you didn't understand? Why?
	Is there something about the course (specific session) that you felt was more relevant to certain groups in the community?
Courses days 4–5	Can you share a story from the course where you learned something new that will help you in an emergency situation?
	Can you share a story from the course where you felt that what you were learning really will not help you in a real emergency situation?
	Can you provide an example about how or what you will do differently given your course learnings?
	What do you think makes a course effective in your community?

## Results

Twenty community members were purposively selected to participate in the course. The results represent the views of the course and research participants alone (n=20).

The findings of this study are grouped into two main themes, the first focused on participant's response to the course personally and as community members, and the second on the community response and context for the course.

### Personal context

Throughout the course, stories about personal experiences with injury, illness or death were shared. Stories were used as a way to learn and the course included scenarios.

The scenarios that we did kind of made me realize that it could actually happen to me if I need to help someone. The reality set in for me especially when we were doing the scenarios. When I did first response [training in the past] it didn't really click in with me it didn't feel like it was going to happen. It's not real. This course was set in an environment that it might happen. (Participant) [Day 5, Sharing Circle]

To many course participants, scenarios and course material also brought forth memories of past experiences.

Doing CPR on baby reminded me of when they did this on my 2 month old granddaughter. They took her and were doing it on the washing machine. I didn't know how to do that. (Participant) [Day 1, Participant Observation]

In contrast to the panic described in previous experiences with injury, illness or death some noted that course participation influenced their confidence.

I just want to thank everybody for participating, thank the instructors a lot: we have 3 doctors present here today and a researcher. I want to thank them for coming and giving us I guess the confidence for us to know what to do in emergency situations. I have boosted up my confidence level if I should see an emergency I'm capable of what I can do. (Participant) [Day 5 Sharing Circle]

Course participants shared stories with underlying messages of self-sufficiency and independence.

Everyone goes out alone. That's what happened to my grandpa once. He dislocated his [points to hip] he was out in the cabin … he managed to crawl out to his boat, crawl up the hill.

(Participant) [Day 3 Focus Group]

However, participants noted informal ways to keep track of individuals when they go into the bush (wilderness), as well as informal reinforcements and support during a crisis. Participants described how when groups go into the bush, there are informal systems in place, such as having an estimated time of return, to ensure safety.

Participant 1: personally I know that I have this knowledge … what I just learned this past week and if I were somewhere if somebody did get hurt or get sick I would know what to do. And what is that stable and unstable.

Participant 2: It's the same in other communities. (Researcher asks: Why?) I think we're just being taught the same ….

Participant 3: The upbringings … the teachings of our parents and grandparents. Be self sufficient in all aspects of life.

[Day 5 discussion with research partners]

Expressions of self-confidence and self-sufficiency were contrasted with descriptions of barriers and challenges in accessing health services and information. A number of participants expressed concerns in accessing care in the community. In the below quote, the participant notes trouble in accessing prescriptions, alluding to prescription opioid abuse and misuse.

To this day I'm scared to get severe headaches again. I went through … it was awful. I was here getting stuck. I saw my face twisting and my eyes …. But I kept going to the nursing station and she told me use hot compresses at home.

That's what they say.

They say you just want a prescription drug.

That's what's stopping the nurses. That's what the abuse is doing to people. You can't even get Tylenol now.

[Day 2 Course, participant observation]

The notion of confidence can also be ascribed beyond the individual to the community, which leads into the second theme group of community context and impact.

### Community context

Participants suggested that knowledge acquired from the course was shared, and that the course provided collective gain.

Participant 1: If they have to sent out a rescue party it will feel that someone with this knowledge will get called upon. We can go out as rescue party. I may forget some stuff. He [referring to another participant] may forget some stuff but together we can share ideas.

Participant 2: I know that there are people spread out across the community. I can call someone closer to respond to that emergency closer to get there. We have to keep this group active.

… .

Participant 3: And the other thing is that (Participant 5) knows the east side [of the outlying areas from the community] and (Participant 1) knows the north side [of the outlying areas from the community] we have people that we can know how to navigate the lake and the river at any time [of the year].

Participant 1: That's the thing with this community that people know the lay out of the land. The territory. Not everyone knows.

Participant 4: It's not just the territory it's the wind, the currents, different parts of the land.

[Group 2, Day 3 Focus Group]

The theme of collective gain and knowledge emerged in many conversations with participants.

Participant 1: When we were doing these scenarios there was some things that we forgot and my partner was there to correct me. He would say what else could be done …. With that way, the way that it came together, I guess he heard what (Participant 2) said we live right across the community. If something happened on the west side the people there who got the training they'd be the first ones. While they are assessing then others could come and see what's not right they could step in and correct and see what's not right.

Participant 2: Team work … ya in each community. The community comes together and works together as one. [Day 5 discussion with research partners)

Course material is meaningful when grounded in the community context. This is a challenge as conventional wilderness first aid courses are developed with the assumption that participants do not live in remote or wilderness areas. Participants were interested in the applicability of the course to their context, which includes periods of time spent in the areas outlying the community, “the bush”. Scenarios were tailored to the community context. Conventional courses employ scenarios where “rescuers” happen upon “victims” who are strangers. In the Sachigo Lake community, there are no strangers.

Participant 1: being outside helps me remember … its more realistic.

Participant 2: Here things happen in any weather, in snow and in blizzards.

[Participant Observation Notes Day 3]

…

We should get someone to jump in the lake for hypothermia … lets make it real.

(Participant) [Participant Observation Notes Day 2]

The research methods and small sample size limit the ability to answer the question of whether the course was effectively tailored to the community. However, participants reminded instructors about their context and questioned its relevance, particularly during the first two days of instruction.

This is a good program but most of the time we're out there with no highways, no nursing stations, so I want some answers around how to take care of people when they are sick. (Participant) [Day 2 Participant Observation Notes]

I don't know what you guys consider wilderness … for us its when we go outside. We don't have a nursing station out there in the bush. (Participant) [Day 3 Participant Observation Notes]

Participants would adapt scenarios to reflect their own experiences. The quote below was in response to a question asking what participants would like to learn more about, suggesting a need for scenarios that commonly occur in the community context.

I had to splint up my daughters’ arm last week. They are rough with each other nowadays. The scenarios involved mostly adults. [Day 3, Focus Group]

Participants reflected on the importance of collaboration on course content.

If you want to learn something it goes both ways. That's the attitude these people have brought here with them. They have something to teach and they have something to learn from us. That's what I appreciate from this one here. (Participant) [Day 3 Focus Group]

## Discussion

The responses to a wilderness first response course in a remote First Nations community were grouped into two main themes, personal and community contexts.

The personal context focused on sharing of stories related to injury and illness, and the ways in which prior experiences to injury influenced individuals. Research has demonstrated that Aboriginal populations in Canada bear a disproportionate burden of trauma and other critical health emergencies in Canada (1,15). While scenarios are an important part of first response teaching and learning, they can bring forth painful recollections of previous experiences.

Empowerment was a significant theme – with participants expressing a strong sense of empowerment from knowledge gained in the course; this contrasted with sentiments of participants related to challenges in accessing health services.

The community context was focused on two themes, the first around the course providing shared community knowledge, with participants’ feeling of being part of a community response to injury. The community context also has implications for pedagogy and highlighted the importance of community input around course content. Grounding content in community context through appropriate scenarios was a means to enhance relevance.

## Limitations

There are a number of limitations to this study related to the practical challenges of conducting participatory action research in remote First Nations communities, as well as the challenges in making general statements based on experiences within one community.

The Sachigo Lake community was approached for this research project in early 2009 through the physician, who shared the idea with the local Health Director, who was a key research partner and champion for this project. The Health Director facilitated access to community resources, selected participants and research partners. The importance of a community research partner who facilitated the collaborative relationship cannot be understated, and may be difficult to replicate. The influence of this research partner and his support for the SLWEREI was well known amongst participants and community members, and this may have influenced what participants expressed, and their abilities to speak freely and critically. The importance of a strong partnership with First Nations community leaders, who then facilitated access and set the stage for meaningful CBPR has been described elsewhere (16).

The role of an embedded evaluator was as much for continuous quality improvement, as it was for on-going feedback and response. Anecdotally, community members suggested that they valued having an evaluation component as it demonstrated flexibility in the curriculum as well as willingness to make real-time changes based on feedback. While the approach used was based on best practices in qualitative research, there was flexibility, including not recording focus groups and having community research partners with little formal research training. In addition, analysis was based on daily researcher coding, which was then validated through conversations with research partners. To follow CBPR and ensure participation throughout the research process, inter-rater reliability was obtained by consulting individuals not present during SLWEREI.

This study is not a programme evaluation but rather a description of a pilot programme in one community. To conduct a comprehensive evaluation on interventions, such as the SLWEREI, there is a need to look at the programme and responses across multiple communities (17).

## Future research

A review of the study's findings suggest that teaching a first aid course in a remote First Nations community should take note of important personal and community contexts.

### Establish strong partnerships and collaboration

Early work to establish partnerships, build trust and ensure a collaborative relationship with the community was a critical building block for this project. An initial site visit helped build trust. Efforts to ensure collaborative partnerships were carried through to the course, where participants expressed the importance of reciprocal learning and exchange.

### Consider previous experiences with injury and critical emergencies

First aid courses in small remote First Nations communities must be sensitive towards the higher burden of injury in these communities and participants’ first-hand experiences.

### Consider how self-sufficiency and self-efficacy are taught and conveyed through course material

Remote First Nations communities like Sachigo Lake are by design, and historically, quite self-sufficient. While conventional first aid courses focus on stabilising patients and preparing them for a hand-off to a more qualified or well-resourced professional, these remote communities may not always have this option available. Teaching course material in a way that is cognizant of the tension between communities’ self-sufficiency and helplessness in accessing external resources is important. Course participants are well aware of barriers to accessing care and course material needs to be grounded in this context.

### Ground course in community context

Conventional first aid courses teach groups of people who work together as a team or who will apply skills as individuals. SLWEREI course participants are neither.

Participants expressed how the course was a way for the whole community to learn and enhance community capacity, rather than individual capacity, in first response. Community members discussed how their knowledge is reinforced by other course participants.

### Utilise appropriate community scenarios and simulations

Scenarios are an important aspect of teaching and learning first aid. Simulations were seen as an important modality for learning and a way to make course material “real”. Community input into developing these scenarios, and feedback around which are effective, is important.

### Fill the research gap

This research is a pilot project. There is a gap in the literature on how a first aid course can build emergency response capacity in remote communities. Conducting a broader evaluation of the course across many communities over time can provide increased depth and breadth to understand key levers to the course’s success or failures, and evaluate impact.

## Conclusions

This article describes how a meaningful research collaboration and partnership was built, fostered and sustained, and how community input and partnership was key to developing and delivering an appropriate and relevant course.

Understanding context is critical to delivering a course that is appropriate and relevant to participants. Teaching content through scenarios and simulations that are community-specific, and recognising how context can be woven into course materials, is important to ensure the course is relevant. Though delivering a first response training programme in a remote First Nations community may not impact morbidity and mortality associated with injury, it has the potential to impact self-efficacy and confidence surrounding first response.

The findings from this pilot study can help provide insights around how an educational intervention in first aid skills can contribute to building capacity in remote communities for managing critical health needs and emergencies.
